# Protocol for a community-based digital storytelling pilot intervention to reduce Hispanic parents’ vaccine hesitancy to immunize their children against COVID-19

**DOI:** 10.1371/journal.pone.0299787

**Published:** 2024-03-19

**Authors:** Alexis Koskan, Linda Larkey, Michael Todd, Sunny Wonsun Kim

**Affiliations:** 1 College of Health Solutions, Arizona State University, Phoenix, Arizona, United States of America; 2 Edson College of Nursing and Health Innovation, Arizona State University, Phoenix, Arizona, United States of America; PLOS: Public Library of Science, UNITED KINGDOM

## Abstract

COVID-19 vaccines, currently available to children over six months old, are a powerful method of reducing the risk of COVID-19-related hospitalizations and death. However, vaccination rates among Hispanic children remain suboptimal, primarily due to parental vaccine hesitancy. Health communication researchers have suggested using culturally aligned storytelling to reduce vaccine hesitancy; however, few studies have evaluated this approach for Hispanic parents of unvaccinated children. Working with community health workers, we will engage Hispanic parents who were previously hesitant to vaccinate their child(ren) against COVID-19 but currently support vaccination. We will ask them to share their stories of conversion in COVID-19 vaccine perspectives to help other parents overcome their mistrust of COVID-19 vaccines. We will then assess the feasibility and acceptability of a web-based pilot digital storytelling intervention based on these conversion stories vs. an information-only control among 80 parents and/or legal guardians of children who are not up-to-date with COVID-19 vaccines. We will also examine pre- to post-intervention changes in vaccine perceptions, hesitancy, intentions, and uptake of children’s COVID-19 vaccination at two months post-intervention. If our pilot study demonstrates feasibility and acceptability while reducing COVID-19 vaccine hesitancy and increasing vaccine uptake, we will conduct a full-scale randomized controlled trial to examine the effectiveness of the DST intervention to reduce vaccine hesitancy.

## Introduction

The American Academy of Pediatrics reported that approximately 15.6 million children in the U.S. tested positive for COVID-19 by the time COVID-19 was no longer being named a pandemic [1]. Although the severity of this virus has decreased, it remains critical to protect children and adolescents against COVID-19 infection and transmission because this virus can lead to prolonged symptoms of COVID-19, ICU admissions, and even death [2], especially among those living with underlying medical conditions such as Type 1 diabetes, obesity, and other chronic conditions [2]. It is imperative to protect the health of all children and adolescents by limiting the spread of COVID-19 and its complications by vaccinating them against the virus [3] and continuing to follow the shifting recommendations for updated vaccine versions and frequency [4].

COVID-19 vaccines are a powerful method of protection against the virus, with up to 95% effectiveness in preventing new infections and reducing COVID-19-related hospitalizations and deaths [5]. However, adherence to the CDC’s COVID-19 vaccine guidelines among children and adolescents has been suboptimal. As of May 10, 2023, rates of receiving at least one COVID-19 vaccine dose were 6% in children ages 6 months to 2 years, 8% of children ages 2–4 years, 39% of 5-11-years-olds, and 71% in 12-17-year-olds [6]. Many parents have chosen not to vaccinate their children against COVID-19 due to their own vaccine hesitancy—the refusal or delayed acceptance of an accessible vaccine [7]. Recent research with low-income Hispanic parents found several reasons for COVID-19 vaccine hesitancy, including a mistaken belief that the COVID-19 vaccine has not undergone enough testing to ensure patient safety, fear of short and long-term side effects (e.g., infertility, neurological effects, impact on children’s developing immune system) [8, 9]; distrust of large societal institutions (government, pharmaceutical industry, and scientists); belief that the vaccine is unnecessary; and worries that the child is too young to receive the vaccine [10]. Many vaccine-hesitant parents expressed a desire to “wait and see” how other children fared after receiving the vaccine before vaccinating their own children [11]. These COVID-19 vaccine (including booster and updated vaccines) concerns could potentially be addressed by other parents’ personal accounts about deciding to vaccinate their child against the virus [9, 11].

Digital storytelling (DST) has shown promise as an effective health promotion strategy [12, 13]. DST, an innovative community-based participatory health communication strategy, empowers individuals to create brief first-person visual narratives (stories) to communicate culturally resonant messages [14]. DST combines digital images, audio recordings, music, and text to document personal experiences [15]. Creating digital stories allows participants to retain control over their own stories [16], engage in open conversations with fellow digital storytellers, share their personal experiences, and discuss the commonalities of these shared experiences with others [17]. Those participating in DST workshops have expressed feeling empowered and have found value in sharing their stories with their communities [18–21], including in the context of COVID-19 vaccine decision-making (among young Black adults) [22]. Digital stories show promise for prompting positive changes in health behaviors [12, 17, 23], and researchers have recommended using this strategy among Hispanic populations [11, 14, 24]. However, little is known about the influence of these storytelling interventions on Hispanic parents’ decisions to vaccinate their children against COVID-19.

Using digital narratives as a vaccine promotion strategy may be more persuasive than relying solely on vaccine data and statistics [25], especially when the stories include scientific information and evoke an emotional response [26]. Prior research has recommended using vaccine stories that illustrate parents’ transformation from vaccine-hesitant to vaccine-accepting to persuade other parents to vaccinate their children [25]. These shared stories about parents’ decision-making process of how they ultimately decided to vaccinate their children against COVID-19 may arouse other parents’ emotional and social responses while delivering important vaccine information. This, in turn, may increase the likelihood of other parents choosing to vaccinate their children. Despite the evident promise, few rigorous studies have assessed the narrative elements for improving COVID-19 vaccine uptake for children, and none have examined theoretically proposed mechanisms for promoting COVID-19 vaccination. The proposed project seeks to develop and refine a novel storytelling approach using digital stories as a culturally grounded intervention for Hispanic parents whose children are eligible for COVID-19 vaccination. Of note, we acknowledge that the cultural/ethnic term “Hispanic” does not represent a singular culture but rather a cluster of many cultures with more or less influence from countries south of the U.S. border (e.g., Mexico and countries in Central and South America) that primarily speak Spanish.

### Conceptual framework

Our DST intervention is guided by a blending of a theoretical framework, the Theory of Planned Behavior (TPB) [27] and a model, Narrative as Culture-Centric Health Promotion [28]. Used together, we suggest that culturally resonant and culturally embedded digital stories that provide examples of the desired health behavior will generate transportation into the story (emotional engagement) and identification with both the character and story. Transportation and identification, in turn, can impact TPB constructs. When applied to COVID-19 vaccination, the TPB posits that individuals who 1) have positive attitudes towards the vaccine, 2) believe others want them to vaccinate their children against COVID-19 (normative beliefs), and 3) are confident in their ability to have their children vaccinated (perceived behavioral control) will intend to vaccinate against COVID-19. Digital stories from formerly vaccine-hesitant parents may address concerns and counterarguments that resonate with other vaccine-hesitant parents and legal guardians, influencing their vaccine attitudes, normative beliefs, and perceived behavioral control. We believe these changes will bolster Hispanic parents’ intentions and behaviors regarding immunizing their children against COVID-19. This paper describes the design of an intervention study to assess the feasibility, acceptability, and preliminary effectiveness of a digital storytelling intervention to reduce COVID-19 vaccine hesitancy among Hispanic parents.

### Study objectives

In this feasibility study, our specific aims include:

**Aim 1:** Develop digital stories (n = 10; each story lasting 2–3 minutes) in a DST workshop with a diverse sample of Hispanic parents/ legal guardians converted from being COVID-19 vaccine-hesitant to vaccine-accepting.

**Aim 2:** Assess the feasibility and acceptability of a pilot web-based DST intervention vs. an information-only control among Hispanic parents and legal guardians (n = 80) of children who are not up to date with CDC-recommended COVID-19 vaccine doses.

**Exploratory aim:** We will explore intervention and control group participants’ patterns of pre- to post-intervention change in vaccine uptake perceptions, vaccine hesitancy, intentions to vaccinate children against COVID-19, and children’s vaccine uptake at two months post-intervention.

## Materials and methods

This proposed pilot study will be conducted in two phases: (a) a formative phase to develop a DST intervention (Aim 1) and (b) a pilot 2-arm randomized controlled trial, assessing feasibility and acceptability (Aim 2) and exploring patterns of change in outcomes for a DST intervention condition and an information control group.

In the first phase of the study, a community advisory board (CAB), comprised of community advocacy groups, leaders of the local Latino population, healthcare providers, maternal and child health experts, and Hispanic parents of children vaccinated against COVID-19, will be actively involved in the formative process to guide the development of stories, and then evaluate, refine, and if needed, update intervention materials. First, the CAB will help identify the types of stories that we hope to select, which ideally will be relevant to Hispanic families’ reasons for COVID-19 vaccine hesitancy. This set of guidelines for types of stories will be used to help guide the DST workshop and assure that the prompts and design features of the digital stories created reflect and contain content and characteristics desired for stories to be used in the intervention.

After the ten digital stories are created, the CAB will help us select the four most compelling stories to use as intervention materials in the second phase of the study. In Phase 2, Aim 2, using a pilot two-arm randomized controlled trial design, we will assess the feasibility and acceptability of a DST intervention compared to an information-only control group. See S1 Fig for the study timeline. The SPIRIT-13 checklist (S1 Checklist) guides this study protocol for a feasibility study. This study received IRB approval from a large public university in the Phoenix Metropolitan area (STUDY00017735).

### Phase 1: Creating digital stories

#### Target population, inclusion, and exclusion criteria

The eligibility criteria for Aim 1, the creation of digital stories, includes the following: ten Hispanic parents or legal guardians who meet the following inclusion criteria: (1) ≥18 years old, (2) Hispanic, (3) a biological parent or a legal guardian (e.g., grandmother) of ≥1 child under 18 years old, and (4) previously hesitant to vaccinate their child (or children) but ultimately decided to have at least one of them vaccinated against COVID-19. English fluency is not required, given the bilingual/bicultural resources of our team. If parents and legal guardians do not meet inclusion criteria or are unwilling to provide informed consent (S1 File), we will exclude them from participating in the digital storytelling workshop.

#### Recruitment and retention

Working with bilingual and bicultural community health workers (CHWs), we will recruit a diverse sample of ten Hispanic parents or legal guardians who meet inclusion criteria in the Greater Phoenix Metropolitan area via word of mouth, community-based organizations, clinics and doctors’ offices, newspapers, and flyers posted in local supermarkets, schools, and churches. CHWs will collect and document formerly vaccine-hesitant parents’ stories about why they become more willing to vaccinate their children. They will also document the age of potential participants’ children. The CHWs will share this information with the study team. The study team and CHWs will meet with the CAB to help select ten parents and/or legal guardians to participate in the digital storytelling workshop (with two additional names listed as backup in case the selected participants decline consent). They will select a stratified sample of adults (based on the child’s age and parents’ preferred language) to include diverse perspectives of parental vaccine perceptions. All study materials will be created in both English and Spanish, and bilingual CHWs will be involved throughout this phase to assist with research activities and to serve as interpreters when needed.

Selected parents/guardians will be re-contacted, and we will conduct informed consent (S2 File) procedures over the phone. For those consented, the workshop will be scheduled to meet the needs of the ten selected participants. To help with study retention, we will offer the participants the storytelling workshop free of charge. These workshops range in price from $400-$1000. Additionally, we will compensate participants $150 per day (for two days) for sharing their time and experience with the research team. We will provide vouchers for childcare for all participants.

#### Setting

Working with our CHWs, we will select a community site that is convenient for digital storytelling participants to conduct this in-person workshop. A certified DST workshop facilitator (name blinded for manuscript submission) will conduct the workshops for two consecutive six-hour days at the selected community site. Our bilingual, bicultural CHWs will attend this two-day workshop to help translate and work with participants as needed.

#### Procedures

We will execute planned activities through in-person workshops (n = 2, one in English and one in Spanish) to guide our participants through the process of crafting 2–3-minute-long digital stories. DST workshops comprise three levels of engagement: (1) an individual process, (2) a group process, and (3) a process co-mediated by participants, researchers, and facilitators to produce a digital story for each participant. The stories will be based on these parents’ experiences of going through the process of transforming their perspectives about COVID-19 vaccines from vaccine-hesitant to vaccine-accepting. The emphasis will be on parents’ experiences, including their initial doubts and concerns about COVID-19 vaccines and overcoming personal belief barriers and vaccine hesitancy. We will record and transcribe all activities to enhance research transparency and rigor.

Questions and prompts based on our prior work (and feedback from our CHWs to ensure their linguistic and cultural competency) will be used in the *individual process* to help Hispanic participants think through and write down their thoughts and feelings, remembering their processes and experiences. We will further use these questions to guide discussions about vaccine hesitancy and to help participants create ideas of the type of story they wish to share and then suggest that their individual initial story “drafts” be shared with the group and receive feedback (*group process*). Reminders to include elements important to them, culturally relevant points, and key information and facts that shifted their perspectives about vaccination will be used to hone in on essential story features. At the end of the first day, participants will be encouraged to go home with the key messages from their stories and choose photos, personal items, or images to bring the next day to incorporate into their storyline.

The second day of the workshop will incorporate the third “process”, the *co-mediation by participants*, *researchers*, *and facilitators* to produce a digital story for each participant. We will collaborate with participants to choose meaningful photos/images, identify story content within their selected pictures, and use software (e.g., Adobe Premier) to create storyboards that combine the stories with the pictures they chose. Participants will incorporate peer input from the “story circle” activity into the final scripts. A university media specialist will help participants record voiceovers. We will add a title, credits, textual graphics, and preferred background music to finalize their stories. In addition, we will provide coaching, technical support, and assistance during the entire process to maximize their learning about digital editing, reduce their frustration, and ensure the stories are personal and authentic.

The ten produced digital stories will reflect participants’ experiences of their children’s COVID-19 vaccination. Our CAB will review all ten stories and select the four most persuasive, culturally relevant, and fact-enriched examples to use in the intervention implemented in Phase 2. CAB members will complete a Narrative Quality Assessment tool [29] immediately after reviewing each story and help select the top four stories that will be used in the intervention.

#### Qualitative data analysis

The focus group discussions with the digital storytellers will be digitally recorded, transcribed verbatim, and, for the Spanish-language focus group, translated into English. To analyze the qualitative data, using an inductive approach, the two lead investigators will read all focus group transcripts and serve as the primary codebook creator and editor, expanding and merging codes as needed [30]. To enhance the rigor of the qualitative analysis, we will conduct a team-based thematic analysis to code the focus group data [31]. One lead investigator (initials provided later) will train a study research assistant in qualitative analysis, and they will meet weekly to reach a consensus on coding focus group data. They will code the focus group data separately and meet to compare codes, reach a consensus on any coding discrepancies, synthesize the data, and analyze findings [30, 31]. They will aggregate the ATLAS.ti output and summarize results for each code before creating a comprehensive summary of the findings.

### Phase 2: Pilot testing the digital stories

*Sample Size*: Aim 2 is to develop and examine the feasibility and acceptability of the DST intervention. To address this aim, we selected a pilot RCT sample size (n = 40 per arm) to provide sufficient opportunity to assess procedures (e.g., recruitment techniques) and protocols (e.g., intervention delivery, assessment methods, data management). Using pilot studies to test intervention efficacy is regarded as inappropriate, so inferential tests of these effects are not proposed. Thus, statistical power was not a consideration in selecting the sample size for this study. The selected sample size should allow us to obtain estimates of standard deviations that can inform sample size calculations for the subsequent fully powered RCT. To ensure balance across study conditions, we will use a covariate-adaptive randomization approach with 4 strata defined by the child’s age group (5–11 years, 12–17 years old) and sex.

#### Eligibility criteria

For Phase 2, examining the feasibility and acceptability of the intervention, we will recruit parents who meet the following inclusion criteria: ≥18 years old, Hispanic, a biological parent or a legal guardian (e.g., grandmother) of ≥1 child aged 5–17 years old who has not received the COVID-19 vaccine, agrees to send and receive study text messages for T3 data collection.

#### Recruitment and retention

Working with our CHWs, we will identify 80 Hispanic parents whose children are not up to date with their COVID-19 vaccinations. We will ask participants to refer others to participate in the study (snowball sampling). Additionally, our CAB will help promote the study within their networks. To promote study retention, we will employ the following strategies to retain participants: maintain a strong rapport with participants at baseline and provide clear explanations of follow-up plans; provide incentives at all assessments to promote long-term participation; and explain the scientific importance of their participation. We will also send participants two reminders via text message, email, or phone before the two-month follow-up, as suggested by the CAB and prior research [12].

### Intervention and control condition procedures

CHWs will send participants a link (via email or text) to the T1 assessment, administered online using the REDCap data collection and management platform. Before participants can begin the study, they will be asked to signify consent. If participants require assistance with the assessment or intervention materials, CHWs may use an iPad to guide participants through them, including reading the survey items to them. After participants complete the T1 assessment, we will randomize them to either the intervention or control group. To increase the robustness of the study design and results, staff who perform the assessments and investigators who handle data and perform analyses will be blinded to the randomized arm assignments. Participants will be recruited to the study that is promoted as “Promoting COVID-19 vaccination” and described as providing generic support without mentioning storytelling content. CHWs will be unblinded because the reminder to view the DST intervention materials is likely to generate conversation revealing the study arm. Data will be entered by blinded staff and identified only by participant ID code.

We will show the intervention group participants the four selected digital stories in randomly assigned, counterbalanced orders. Immediately after viewing each story, intervention participants will be asked to rate the story using the Narrative Quality Assessment tool [29]. This tool asks participants to rate the degree to which they identify with the story narrator and the story. After participants have watched all four stories and completed their corresponding Narrative Quality Assessment, we will ask them to complete the T2 assessment. This survey asks participants about their COVID-19 vaccine perspectives.

After completing the T1 assessment, control group participants will receive a CDC COVID-19 Vaccine Information Sheet for their child’s age. They will be given time to review the document. When they are ready, they will be asked to complete the T2 assessment. All participants (intervention and control) will receive a resource kit containing information about where to obtain vaccines (e.g., walk-in clinics) so they do not need to wait for the child’s annual check-up to receive the vaccine from their regular provider. Two months later, we will contact all participants and ask them to complete a follow-up assessment (T3) to determine if parents had their child(ren) vaccinated against COVID-19. We will compensate all participants $20 for each survey they complete for the study.

Additionally, we will conduct four focus groups (of 6–8 Hispanic parents each) with a sample of intervention group participants. We will recruit a stratified sample of intervention participants, including children who received COVID-19 vaccines and those who remained unvaccinated by T3, to participate in post-intervention focus groups. In these focus groups, we will explore their perceptions of the DST intervention and its impact on their decisions to vaccinate or not vaccinate their children. We will compensate all focus group participants $30.

### Study outcomes

We will assess feasibility of the RCT protocol and procedures with measures of participation rate (% of eligible participants who consent to participate), retention rate (% of T1 participants who provide data at T2 and T3), and involvement (% of participants who view all stories and complete all self-report assessments). Acceptability will be assessed via post intervention group discussions with intervention participants.

#### Measures of proposed mediators and clinical outcomes

*Identification with and transportation by the narrative* will be assessed using the Identification and Transportation subscales of the Narrative Quality Assessment Tool [29]. Each subscale comprises six items (e.g., “I could really relate to the people in the story” for identification” and “The story created a picture in my mind” for transportation) with response options ranging from 1 (*strongly disagree*) to 4 (*strongly agree*), and both have shown good reliability (Cronbach’s α = 0.93 and 0.95 for Identification and Transportation, respectively).

*Parent’s attitudes about vaccinating their child(ren) against COVID-19* will be assessed with a validated six-item measure of vaccine attitudes [32] (α = 0.83) adapted to reflect attitudes toward COVID-19 vaccination for children (e.g., “I believe that getting the COVID-19 vaccine is beneficial for my child.”).

*Parent’s perceived norms about vaccinating their child(ren) against COVID-19* will be assessed using validated six-item measure of perceived norms regarding vaccinations [32] (α = 0.90) adapted to reflect norms regarding COVID-19 vaccination for children (e.g., “My child’s doctor thinks I should have my child vaccinated against COVID-19.”).

*Parent’s perceived behavioral control to vaccinate their child(ren) against COVID-19* will be assessed using a validated four-item measure [32] (α = 0.78) updated to reflect COVID-19 vaccination for children (e.g., “Getting the COVID-19 vaccine for my child is completely up to me.”).

*Parent’s COVID-19 vaccine hesitancy* will be measured at three time points (see Table 1 for assessment timeline) using a modified version of the 15-item Parent Attitudes About Childhood Vaccines (PACV) questionnaire [33]. This questionnaire has three subscales which measures parents’ immunization behaviors (2 items), beliefs about vaccine safety and efficacy (4 items), and general attitudes about vaccines (9 items) All items use a 4-point response scale (1 = strongly disagree to 4 = strongly agree). This measure has shown good reliability (Cronbach’s αs = 0.74, .74, and .84, respectively).

**Table 1:** Phase 2 assessment timeline.

		Time point when computed or collected		
Outcome or construct(s) assessed	Measure	T1	T2	T3
Feasibility	Participation rate	X		
Feasibility	Retention rate		X	X
Feasibility	Involvement		X	
Acceptability	Satisfaction questionnaire (2 items)		X	
Acceptability	Focus group feedback			X
Identification and Transportation	Narrative Quality Assessment Tool (two 6-item subscales) [29]		X	
Vaccine attitudes	Attitudes about vaccinating child against COVID-19 (X items)	X	X	
Perceived vaccine-related norms	Perceived norms about vaccinating child against COVID-19 (X items)	X	X	
Perceived behavioral control	Perceived behavioral control to vaccinate child against COVID-19 (X items)	X	X	
Vaccine hesitancy	Adapted PACV questionnaire (18? 15? items) [check ref]	X	X	X
Vaccine intentions	Intentions to vaccinate child against COVID-19 (X items)	X	X	
Vaccination behavior	Vaccine uptake following T2 (1 item)			X

*Parent’s intentions to vaccinate child(ren) against COVID-19* will be measured using a single previously validated survey item developed based on Theory of Planned Behavior constructs, adapted to reflect COVID-19 vaccination intentions (“I intend to vaccinate my child against COVID-19 in the next two months.”) [32].

*Child’s COVID-19 vaccine uptake* will be measured using two items, with yes/no response options. One item will assess whether the child (or children) has (or have) received 1 or more doses of any COVID-19 vaccine. The other item, asked only of those parents who have had their children vaccinated, will assess whether the child received a COVID-19 vaccine dose since T2.

### Data monitoring

For Phase 2, we will use the REDCap web-based platform for collection and management of quantitative data. REDCap uses a robust firewall system with regular scans for vulnerabilities, stores data on a secured server, and transports data using HIPPA-adherent encryption. De-identified questionnaire data will be downloaded from REDCap to an access-controlled cloud-based storage platform, where they will be checked, cleaned, scored, and saved using SPSS version 28 and R version 4.3. Data quality checks will be conducted throughout the data collection period of Phase 2 and immediately prior to analyses. Once the final datasets have been downloaded, merged, and stored securely, we will delete all identifying information from the project’s REDCap database. We will perform all quantitative analyses and data visualization in SPSS version 28 and R version 4.3.

During Phase 2, the study statistician will monitor and generate reports regarding participant recruitment, enrollment, and retention for the study team and will conduct and oversee interim analyses at least monthly during the data collection period. A Data and Safety Management Board (DSMB) will be established to ensure proper oversight and monitoring of this intervention study and thereby ensure the safety of participants and the fidelity and integrity of the data. The study team will prepare quarterly reports for the DSMB with details of (a) participant recruitment, enrollment, and retention; (b) reasons for participant dropout; and (c) any study-related adverse events experienced by participants. We will stop the study if the lead study investigators and the DSMB identify more harms and risks (e.g., threats to physical and mental health) than benefits for participants.

### Statistical analysis

The central aim of the Phase 2 pilot trial is to assess feasibility and acceptability of the study protocols and procedures. Accordingly, quantitative analyses will focus on comparison of these outcomes to a priori benchmark values. Formal and informal testing of intervention efficacy testing is inappropriate in feasibility studies as is the derivation of effect size estimates [34–37]. Accordingly, examination of data on proposed mediators and clinical outcomes will be limited to exploratory and descriptive analytic procedures and visualization.

#### Feasibility

We will assess feasibility by comparing the observed participation rate (percentage of eligible individuals agreeing to participate), retention rate (proportion retained through follow-up), and involvement to *a priori* benchmarks (80% for each of the three indicators). We will also examine between-group differences in rates of retention and missing data at T2 and T3.

#### Acceptability

We will assess acceptability using multiple information sources. First, we will determine intervention satisfaction using two questionnaire items previously used in our DST intervention research (“I am satisfied with the program” and “I will recommend this program to others”), with response options ranging from 1(*Strongly Disagree*) to 5 (*Strongly Agree*). A mean satisfaction score ≥4.0 indicates that the intervention is acceptable. In addition, we will pilot test a post-intervention group discussion guide that study investigators created to explore invention acceptability with two CAB members. The current draft guide asks intervention participants about their overall thoughts related to participating in the interventions, watching other parents’ digital stories, and identifying stories they personally relate to and others they find less relevant. We also will ask parents about their acceptability of viewing stories about parents’ vaccination decisions and whether they believe other parents would benefit from watching similar vaccine-transformation stories. We will use the approach described in Aim 1 of the study to analyze post-intervention group discussions.

#### Measures of proposed mediators and clinical outcomes

We will use univariate and bivariate statistics (e.g., means, proportions, standard deviations) and plots (e.g., histograms, box plots, scatterplots) to examine distributions of, and associations among, key theoretical mediators (identification and transportation subscale scores, measures of TPB constructs), vaccine hesitancy, vaccination intentions, and vaccination behavior to examine patterns of change in these mediators and vaccine hesitancy and rates of vaccine uptake and to explore between-group (intervention vs. control) differences in these patterns. To assess whether participants’ children received any additional COVID-19 vaccine doses, we will explore between-group differences in T3 vaccination rates using 2-way tables.

### Dissemination

We will collaborate with our community partners to develop brief reports for the community. We will use lay language to enhance the understanding of the study findings. We will present the findings via different outlets (e.g., community forums, newsletters, and social media) based on input from community partners and key stakeholders. For the scientific community, we will share study findings via conference presentations and manuscripts submitted for publication in peer-reviewed journals. We will place de-identified data in readily accessible public databases such as ClinicalTrials.gov and Open Science Framework. We will make all efforts to release data by publishing results as quickly as possible and archive de-identified data on PubMed Central within one year of publication.

## Discussion

The proposed research addresses major public health concerns of COVID-19 vaccine hesitancy in racial and ethnic minorities. Our study will provide critical insight into the potential use of stories and what works best in the narrative intervention paradigm to persuade vaccine-hesitant Hispanic parents. Study findings will inform a future full-scale RCT to examine the effectiveness of a culturally embedded DST intervention to reduce COVID-19 vaccine hesitancy in our target population. This more extensive study could be used in future DST interventions to increase childhood and adolescent immunizations (e.g., flu, HPV). In addition, our innovative research may provide evidence of scalable, disseminatable strategies to reduce vaccine hesitancy and can be used for other rapid vaccination efforts among Hispanic families. Results from this effectiveness study could be among the first to illustrate the impact of digital storytelling on improving childhood and adolescent immunizations via a scalable, disseminatable, low-cost strategy to reduce parental vaccine hesitancy for other child and adolescent vaccines or for future outbreaks.

### Limitations and challenges

One challenge can be recruiting Hispanic parents to participate in the digital storytelling workshop and an additional 80 parents to test the intervention materials. To address this challenge, we are working with local community health workers who have previous experience promoting COVID-19 immunizations to help us recruit Hispanic parents to participate in the study. We are also asking our CAB to share the study information with individuals within their networks, many of whom work with additional community-based organizations that are well-connected to local Hispanic communities.

An additional challenge is the influence of other ongoing public health efforts aimed at increasing COVID-19 vaccination. We aim to address this challenge with the timing of our three surveys (T1, T2, T3), which will help us determine the impact of our DST intervention. Post-intervention focus groups will allow us to further explore the effects of our intervention on parents’ vaccine hesitancy and behaviors related to vaccinating their children against COVID-19.

## Supporting information

S1 Checklist SPIRIT 2013 checklist: Recommended items to address in a clinical trial protocol and related documents*. (DOC)

S1 Fig Schedule of enrollment, interventions, and assessments. (DOCX)

S1 File Digital storytelling informed consent. (DOCX)

S2 File Intervention informed consent. (DOCX)

S1 Protocol Study protocol. (DOCX)
