# Supplementary material for: Protocol for a community-based digital storytelling pilot intervention to reduce Hispanic parents’ vaccine hesitancy to immunize their children against COVID-19
Source: PLoS One. 2024 Mar 19;19(3):e0299787. doi: 10.1371/journal.pone.0299787 (PMC10950256; doi:10.1371/journal.pone.0299787)
Supplement: S1 Fig — (DOCX) [file pone.0299787.s002.docx]

Fig S1. Schedule of enrollment, interventions, and assessments

|  | **STUDY PERIOD** | | | | | | | |
| --- | --- | --- | --- | --- | --- | --- | --- | --- |
|  | **Enrolment** | **Allocation** | **Post-allocation** | | | | | **Close-out** |
| **TIMEPOINT**** | ***-t_1_*** | **0** | ***t_1_*** | ***t_2_*** | ***t_3_*** | ***t_4_*** | ***etc.*** | ***t_x_*** |
| **ENROLMENT:** |  |  |  |  |  |  |  |  |
| **Eligibility screen** | X |  |  |  |  |  |  |  |
| **Informed consent** | X |  |  |  |  |  |  |  |
| **Allocation** |  | X |  |  |  |  |  |  |
| **INTERVENTIONS:** |  |  |  |  |  |  |  |  |
| ***Digital Storytelling*** |  |  |  |  |  |  |  |  |
| ***Control Group (Information only)*** |  |  |  |  |  |  |  |  |
| **ASSESSMENTS:** |  |  |  |  |  |  |  |  |
| ***Participation rate*** | X |  | X |  |  |  |  |  |
| ***Vaccine hesitancy,***  ***Vaccine attitudes, Perceived vaccine-related norms, Perceived behavioral control, Vaccine hesitancy, Vaccine intentions*** | X |  | X |  |  |  |  |  |
| ***Vaccine Hesitancy*** |  | X |  | X | X |  |  |  |
| ***Vaccine attitudes, Perceived vaccine-related norms, Perceived behavioral control, Vaccine hesitancy, Vaccine intentions*** |  | X |  | X |  |  |  |  |
| ***Retention rate, Vaccine hesitancy*** |  | X |  | X | X |  | . |  |
| ***Involvement, Satisfaction, Narrative Quality Assessment*** |  | X |  | X |  |  |  |  |
| ***Intervention Acceptability, Vaccination Behavior*** |  | X |  |  | X |  |  | X |

*Recommended content can be displayed using various schematic formats. See SPIRIT 2013 Explanation and Elaboration for examples from protocols.

**List specific timepoints in this row.
