# Supplementary material for: Protocol for a community-based digital storytelling pilot intervention to reduce Hispanic parents’ vaccine hesitancy to immunize their children against COVID-19
Source: PLoS One. 2024 Mar 19;19(3):e0299787. doi: 10.1371/journal.pone.0299787 (PMC10950256; doi:10.1371/journal.pone.0299787)
Supplement: S1 File — (DOCX) [file pone.0299787.s003.docx]

**S3:** Digital Storytelling Informed Consent

***Title of research study:*** Reducing Vaccine Hesitancy among Hispanic Parents of COVID-19 Vaccine-Eligible Children

***Investigators:*** NAMES

Arizona State University, College of Health Solutions

***Why am I being invited to take part in a research study?***

We are inviting you to participate in a project about COVID-19 vaccination. To participate in this study, you must meet the following inclusion criteria:

(1) 18 years old or older

(2) self-identify as Hispanic

(3) a biological parent or a legal guardian of at least one child under 18 years old, and

(4) was previously hesitant to vaccinate their child but ultimately decided to have them vaccinated against COVID-19.

This research, led by ASU professors NAMES, aims to create a series of personal stories that illustrate how parents changed their minds and decided to vaccinate their children against COVID-19. The stories will be presented to other Hispanic parents and legal guardians like you to help them decide if they should have their child receive the vaccine. We are asking you to help us create one of these stories because, at one time, you were hesitant to vaccinate your child against COVID-19 but changed your mind and had them vaccinated. We are also asking you to complete a Story Release Form so that we can use your digital story for this study.

***Why is this research being done?***

Among children and adolescents, infection with COVID-19 can lead to health complications, hospitalizations, and death. COVID-19 vaccines are available to children over six months, and these vaccines are powerful tools against this catastrophic pandemic. However, in most southwestern U.S. states, Hispanic children have the lowest COVID-19 vaccination rates among children. Lower vaccination rates in children are primarily due to parental vaccine hesitancy. Our team of health communication and public health experts want to work with you to create a digital storytelling intervention to help reduce other parents’ COVID-19 vaccine hesitancy and increase their children’s vaccine uptake.

***How long will the research last?***

This research will last two days. Each day, we will meet for up to eight hours.

***How many people will be studied?***

Ten parents and legal guardians will participate in creating digital stories.

***What happens if I say yes, I want to be in this research?***

It is up to you to decide whether or not you will participate in this study. Participation is voluntary.

We will carry out all planned activities through in-person workshops to help you create your video-recorded digital story (each 2-3 minutes long). A certified digital storytelling workshop facilitator, NAME, will conduct the workshops for two consecutive 8-hour days. The stories will be based on your experiences and processes for deciding to vaccinate your child against COVID-19. The emphasis will be on your experiences, including doubts, concerns about COVID-19 vaccines, and overcoming personal belief barriers and vaccine hesitancy. We will video-record the online workshop.

Story idea generation: We have created questions and prompts based on our prior work (e.g., What led you to have your child vaccinated?) to help you think through and write down your thoughts and feelings, remember your processes and experiences, and share them with the group -about how you changed your mind and decided to vaccinate your child against COVID-19. Next, we will facilitate a virtual story circle using scripts you generated in the “story idea generation” activity. Participants will be encouraged to share their stories with the group and receive feedback.

On the second day of the workshop, we will work with you to choose meaningful photos/images, identify story content within your selected pictures, and use software (e.g., Adobe Premier) to create storyboards that combine the stories with the pictures you have chosen. All photos used in the videos will only have images of you, the participant. Do not photos of other individuals with their identifying images in these pictures. We will help you incorporate peer input from the “story circle” activity into the final story scripts. We will work with you to audio record voiceovers. To finalize your stories, we will add a title, credits, textual graphics, and preferred background music. In addition, we will provide coaching, technical support, and assistance during the entire process to maximize your learning about digital editing and ensure the video-recorded stories are personal and authentic.

Workshop evaluation: After the workshop, we will facilitate a group discussion to better understand your experiences and capture any experiences not included in the stories. In this discussion, we will use questions (e.g., What was your overall experience during the workshop?”) to guide the improvement of future digital storytelling workshops. We will audio record this discussion.

***What happens if I say yes, but I change my mind later?***

You can leave the research at any time it will not be held against you. If you decide to leave the research, please contact the investigator (NAME, 480-884-2533, Extension 42533) so that she can remove your study information (e.g. demographic information, remove digital story).

***Is there any way being in this study could be bad for me?***

Given that the topic of COVID-19 vaccines has been a polarizing health issue, telling personal stories or viewing videos that present stories about COVID-19 vaccines may make participants feel anxious.

Further, in group activities such as this digital storytelling workshop and the workshop evaluation discussion, there is a risk that you will not remain anonymous and that your responses will not remain confidential. This is a risk with research conducted with groups of people.

***What happens to the information collected for the research?***

Your privacy is of utmost importance to the researchers of this study. The following steps will be taken to make sure your personal information and identity are protected.

- The researcher will keep all study materials locked in a secure location.
- Only the research team will have access to your identifiable information. They will not keep any paper documents with this information.
- To keep your information confidential, we will assign you a study ID number when you enroll in the study. We will keep a master list of your ID numbers and identifying information (name, contact information, study ID number) separately in a secured, locked database on a password-protected computer kept in a locked office. We will keep your identifying information in this master list until after we use and test the digital stories in the second part of our study. After we use the stories, we will destroy all identifying information we have for you.
- We will keep your digital story for up to five years after the study.
- You can withdraw from the study at any time before we test the digital stories with up to 80 Hispanic parents of COVID-19 vaccine-age-eligible children. After creating the digital stories, if you change your mind and do not want to allow us to use your story, please contact NAME (480-884-2533, Extension 42533) at your earliest convenience.
- Only the study team will see the transcribed electronic version of the post-workshop discussion. This transcript will not have any of your personal information in it.
- At the conclusion of the study, the researchers may publish their findings. The information will be presented in aggregate form, and you will not be able to be identified in any publications or presentations.
- De-identified data collected as a part of the current study will not be shared with others (e.g., investigators or industry partners) for future research purposes or other uses.

***What else do I need to know?***

This research is being funded by Arizona State University’s Southwest Interdisciplinary Research Center. If you agree to participate in this interview, we will give you a $300 Tango e-gift card (where you can choose where to spend that e-gift card) after completing this workshop.

***Who can I talk to?***

If you have questions, concerns, or complaints, or think the research has hurt you, talk to the research team *at* **480-884-2533, Extension 42533 (Name, one of the study leads).**

This research has been reviewed and approved by the Bioscience IRB (“IRB”). You may talk to them at (480) 965-6788 or research.integrity@asu.edu if:

1. Your questions, concerns, or complaints are not being answered by the research team.

You cannot reach the research team.

You want to talk to someone besides the research team.

You have questions about your rights as a research participant.

You want to get information or provide input about this research.

Please let me know if you want to participate in this **digital storytelling** workshop.

Signature of Participant: ________________________________ Date: ___________

Please also sign below if you are willing to participate in a **focus group discussion** (workshop evaluation) immediately after the workshop ends on Day 2.

Signature of Participant: ________________________________ Date: ___________

**INVESTIGATOR’S STATEMENT**

“I certify that I have explained to the above individual the nature and purpose, the potential benefits and possible risks associated with participation in this research study, have answered any questions that have been raised, and have witnessed the above signature. These elements of Informed Consent conform to the Assurance given by Arizona State University to the Office for Human Research Protections to protect the rights of human subjects. I have provided (offered) the subject/participant a copy of this signed consent.”

Signature of Investigator: ________________________________ Date: ___________
