# Supplementary material for: Protocol for a community-based digital storytelling pilot intervention to reduce Hispanic parents’ vaccine hesitancy to immunize their children against COVID-19
Source: PLoS One. 2024 Mar 19;19(3):e0299787. doi: 10.1371/journal.pone.0299787 (PMC10950256; doi:10.1371/journal.pone.0299787)
Supplement: S2 File — (DOCX) [file pone.0299787.s004.docx]

**S4:** Intervention Informed Consent

**STUDY TITLE: Reducing Vaccine Hesitancy among Hispanic Parents of COVID-19 Vaccine-Eligible Children**

Currently, we are conducting a research study to explore your experiences viewing digital stories about Hispanic parents’ decisions to vaccinate their children against COVID-19.

We are inviting you to participate in this study because you watched four digital stories about parents who decided to vaccinate their children against COVID-19. Study participation involves taking part in a one-time audio-recorded focus group estimated to last up to 60 minutes. You have the right not to answer any question and to stop participation at any time.

You must be 18 or older to participate in the study and have participated in watching digital stories two months ago for this same study. Your participation in this focus group is completely voluntary. If you choose not to participate or to withdraw from the study at any time, there will be no penalty. All participants will receive a $25 e-gift card for volunteering to participate in this part of the study. Otherwise, there are no other benefits to taking part in the study.

There are a few risks associated with this study. First, you may experience discomfort when answering questions about your COVID-19 vaccine perceptions and behaviors. You can skip any question you do not want to answer and stop participating anytime. Additionally, with focus group participants only, there is a risk that you will not remain anonymous and that your responses will not remain confidential. This is a risk with research conducted with groups of people. The results of this study may be used in reports, presentations, or publications. However, your name will not be used, and all study results will be shared in aggregate form.

We are also asking your permission to audio record the focus group. Only the research team will have access to the recording. The recording will be deleted immediately after being transcribed and any published quotes will be anonymous. To protect your identity, please refrain from using names or other identifying information during the focus group. Let us know if, at any time, you do not want to be recorded, and I will stop.

If you have any questions concerning the research study, please contact one of the study’s Principal Investigators, NAMES AND CONTACT INFORMATION.

If you have any questions about your rights as a subject/participant in this research, or if you feel you have been placed at risk, you can contact the Chair of the Human Subjects Institutional Review Board, through the ASU Office of Research Integrity and Assurance, at (480) 965-6788.

By signing below, you are agreeing to be part of the study.

Name: ______________________________________

Signature: ____________________________________ Date: ________________
