# Supplementary material for: Protocol for a community-based digital storytelling pilot intervention to reduce Hispanic parents’ vaccine hesitancy to immunize their children against COVID-19
Source: PLoS One. 2024 Mar 19;19(3):e0299787. doi: 10.1371/journal.pone.0299787 (PMC10950256; doi:10.1371/journal.pone.0299787)
Supplement: S1 Protocol — (DOCX) [file pone.0299787.s005.docx]

**S5: Study Protocol**

| Protocol Title  Reducing Vaccine Hesitancy among Hispanic Parents of COVID-19 Vaccine-Eligible Children | |
| --- | --- |
| 1. **Background and Objectives** | |
| Among children and adolescents, infection with SARS-CoV-2 (COVID-19) can lead to health complications (e.g., multisystem inflammatory syndrome, long COVID), hospitalizations, and death.^1^ COVID-19 vaccines are available to children over six months, and these vaccines are powerful tools against this catastrophic pandemic.^2^ However, Hispanic/Latino children have lower COVID-19 vaccination rates than White non-Hispanic children.^3^ In most southwestern U.S. states, Hispanic children have the lowest COVID-19 vaccination rates among pediatric populations.^4^ Lower vaccination rates in children are primarily due to parental vaccine hesitancy.^5^ Considerably more work is needed to decrease parental COVID-19 vaccine hesitancy among Hispanic parents. Our team of health communication and public health experts proposes a community-based theory-driven intervention that utilizes culturally-grounded narratives from digital storytelling to reduce Hispanic parents’ COVID-19 vaccine hesitancy and increase their children’s vaccine uptake.  Narrative-based interventions are powerful tools for persuading individuals to enact health behaviors (vaccination) that require an immediate personal cost (discomfort) for a longer-term gain (disease immunity).^6, 7^ Our prior work (see Section C.3) using DST to improve vaccine uptake has demonstrated that emotional engagement with digital stories can promote vaccine-related behavior.^8^ Our current study will examine digital storytelling (DST), a specific form of culturally-grounded narrative developed via community engagement,^8-10^ to reduce Hispanic parents’ COVID-19 vaccine hesitancy. No research, to our knowledge, has used digital stories to decrease Hispanic parents’ vaccine hesitancy. Therefore, it is critical to assess which stories resonate with and are most persuasive for those who are hesitant to have their children receive COVID-19 doses and then explore the impact of an intervention utilizing these stories on parents’ decisions to vaccinate their children against COVID-19.  Guided by the Theory of Planned Behavior (TPB)^11^ and storytelling as culture-centric health promotion,^12^ our study will assist Hispanic parents and legal guardians who were formerly COVID-19 vaccine-hesitant create digital stories to share how and why their vaccine perceptions changed. They will share their reasons for converting from COVID-19 vaccine-hesitant to being willing to vaccinate their child(ren). Based on our combined theories, these culturally resonant stories, drawn from other community members’ lived experiences, will generate the experience of *transportation* (emotional engagement) and *identification* (with characters, stories, and cultural elements) and will influence viewers’ attitudes, perceptions of social norms, beliefs, intentions, and behaviors relevant to vaccination completion. To further inform and refine our DST intervention, a community advisory board (CAB) comprising key stakeholders will help identify the most persuasive, influential, and culturally relevant stories. In addition, the CAB will select the top four stories to test the influence of these stories on Hispanic parents’ willingness to vaccinate their children against COVID-19. Then, we will test the use of these stories in a pilot randomized controlled trial (RCT). Collaborating with local community health workers (CHWs), we will recruit 80 vaccine-hesitant Hispanic parents to participate in a pilot 2-arm RCT with a pre-test and post-test design. Control arm participants (n=40) will receive information only. Finally, we will examine our study’s feasibility and acceptability via focus groups (n=4) with intervention participants.  **Specific Aims:**  **Aim 1**: Develop one digital story per participant (n=10; each story lasting 2-3 minutes) in a DST workshop with a sample of Hispanic parents/ legal guardians converted from being COVID-19 vaccine-hesitant to vaccine-accepting.  **Aim 2:** Assess the feasibility and acceptability of a web-based pilot DST intervention vs. an information-only control among Hispanic parents and legal guardians (n=80) of children who are not up-to-date with CDC-recommended COVID-19 vaccine doses.  **Exploratory aim:** We will explore intervention and control group participants’ (n=80) patterns of pre- to post-intervention change in vaccine uptake perceptions, vaccine hesitancy, intentions to vaccinate children against COVID-19, and children’s vaccine uptake at two months post-intervention. | |
| **Data Use** |  |
| Data will be published in peer-reviewed journals and presented at academic conferences | |
| **Inclusion and Exclusion Criteria** | |
| We have the following inclusion criteria for Aim 1 participants (n=10): (1) is ≥18 years old, (2) self-identifies as Hispanic, (3) is a biological parent or a legal guardian of at least one child under 18 years old, and (4) was previously hesitant to vaccinate their child but ultimately decided to have them vaccinated against COVID-19.  The inclusion criteria for participants in Aim 2 (n=80) include: (1) is ≥18 years old, (2) self-identifies as Hispanic, (3) is a biological parent or a legal guardian of at least one child under 18 years old; (3) their child(ren) are not up-to-date with COVID-19 vaccine doses; and (4) agrees to send and receive a text message and submit a photo of their child’s immunization record for T3 data collection two months after they participated in viewing the digital stories  **English fluency is not required, given the bilingual/bicultural resources of our team. We will exclude individuals who do not meet inclusion criteria or are unable/ unwilling to provide consent. | |
| **Number of Participants** | |
| In Aim 1, 10 Hispanic parents/legal guardians will be recruited into the study.  In Aim 2, 80 participants will be enrolled in the study; 40 in the control group / 40 in the intervention group  Total participant = 90 | |
| **Recruitment Methods** | |
| We have assembled a **Community Advisory Board (CAB)** to support this research, representing immunization advocacy groups, pediatric practitioners, and Hispanic parents of COVID-19 vaccine-age-eligible children, including those who were previously vaccine-hesitant. MPI Koskan and Kim have experience building and managing similar CABs and working on Hispanic population-based coalitions. They will continue calling relevant organizations and identifying local leaders via snowball sampling to expand this multi-stakeholder panel.  To encourage the CAB to remain active in the study (retention), we will meet quarterly and provide flexible meeting dates using Doodle Poll software. All CAB meetings will be held online via Zoom or in-person (will ask preference of CAB), and we will record the meetings. If members are experiencing technical difficulties or issues with internet access, they may attend the Zoom meeting by calling in as a conference call. In addition, we will share study updates with our CAB via monthly newsletters to report on the study’s progress. We will provide incentives to CAB members for volunteering their time and sharing expertise in this study. We have budgeted CAB incentives which include $50/hr per person for 2 hours in Year 1 of the study (to help identify digital stories for the intervention and 1 hour in Year 2 ($50 x 10 people) to disseminate study outcomes of the research.  **Aim 1:** Creating Digital Stories  **Recruitment of 10 Hispanic Parents/Legal Guardians:**   - We will recruit a total of ten Hispanic parents and legal guardians who report previously being hesitant to vaccinate their children (under age 18) against COVID-19 but who ultimately decided to immunize them against this virus. - We will work with our three community health workers (CHWs) to identify local community members interested in this opportunity. Our CHWs will recruit from various community-based venues such as churches and schools.   Retention:   - For study retention, we are offering participants in Study Aim 1 a free digital storytelling workshop offered over two consecutive eight-hour days. Costs to attend these workshops range from $395-$1000. - We will also compensate participants for their time ($150 per day for two days, a total of $300) and provide refreshments/parking/vouchers for childcare. - We will share their final digital narratives with them via a USB drive.   **Aim 2** and the Exploratory Aim: Exploring the feasibility and general impact of DST intervention  **Recruitment of Participants for the Online Intervention**   - We will work with our CHWs to identify 80 Hispanic parents whose children are not up-to-date with their COVID-19 vaccinations. - We will ask participants to refer others to participate in the study (snowball sampling).   Retention: We will employ proven strategies to retain participants, including the following:   - Maintaining a strong rapport with participants at baseline and providing clear explanations of follow-up plans; - Providing incentives at all assessments to promote long-term participation; - Explaining the scientific importance of their participation; and - Sending two reminders via text message, email, or phone before the two-month follow-up as suggested by the CAB and prior research. | |
| **Study Timelines** | |
| We estimate that the proposed project will take two years, from start-up to dissemination of findings. We will hire/train staff and obtain IRB approval in months 1-3. Participant recruitment, DST workshops, intervention delivery, and data collection will occur in months 4-20. The final months allow sufficient time to analyze data and disseminate results via professional and community outlets. | |
| **Procedures Involved** | |
| We will develop a digital storytelling (DST) intervention and conduct a pilot two-arm randomized controlled trial, using a pre-and post-test design, collecting data at three time points (baseline, immediate post-intervention, and two-months post-intervention).  **Aim 1** Sample: Working with community health workers, we will recruit a diverse sample of ten Hispanic parents or legal guardians who meet inclusion criteria. If parents and legal guardians do not meet inclusion criteria or are unwilling to provide informed consent, we will exclude them from participating in the digital story workshop (to create the stories).  Number of stories: Evidence suggests that the optimal number of DST workshop participants is 8-10 people, resulting in 10 individual stories. Therefore, our study will initially include ten digital stories.  Setting: We will execute planned activities through in-person workshops to guide our participants to develop digital stories (each 2-3 minutes long) through a process of ideation, development, production, post-production, and digital distribution. A certified DST workshop facilitator, MPI Kim, will conduct the online workshops for two consecutive 8-hour days. Procedures: DST comprises three components: (1) an individual process, (2) a group process, and (3) a process co-mediated by participants, researchers, and facilitators. The stories will be based on these parents’ experiences of going through the process of transforming their perspectives about COVID-19 vaccines from vaccine-hesitant to vaccine-accepting. The emphasis will be on parents’ experiences, including doubts, concerns about COVID-19 vaccines and overcoming personal belief barriers and vaccine hesitancy. We will digitally record and transcribe all activities to enhance research transparency and rigor.  We will conduct the following activities to address Aim 1:  Story idea generation: We created questions and prompts based on CAB input and our prior work (e.g., What led you to have your child vaccinated?) to help Hispanic participants think through and write down their thoughts and feelings, remember their processes and experiences, and share them with the group. This approach allows participants to comfortably express their ideas and is effective when working with low-literacy populations.  Story Circle: Next, we will facilitate a virtual story circle using the initial scripts generated in the “story idea generation” activity. This step will support participants in elaborating and interpreting script meanings related to paradigms of vaccination and prevention for their children. Participants will be encouraged to share their stories with the group and receive feedback.  Visualizing activity: On the second day of the workshop, we will collaborate with participants to choose meaningful photos/images, identify story content within their selected pictures, and use software (e.g., Adobe Premier) to create storyboards that combine the stories with the pictures they chose. All photos used in the videos will only have images of the participant who signed the release form. Participants will be informed that they may not include other individuals with their identifying images in these pictures.  Video recording & editing: Participants will incorporate peer input from the “story circle” activity into the final story scripts. A university media specialist (from MPI Koskan’s academic department) will help participants record voiceovers. To finalize their stories, they will add a title, credits, textual graphics, and preferred background music. In addition, we will provide coaching, technical support, and assistance during the entire process to maximize their learning about digital editing, reduce their frustration, and ensure the stories are personal and authentic.  Workshop evaluation: After the workshop, we will facilitate a group discussion to better understand participants’ experiences and capture any experiences not included in the stories. In this recorded discussion (treated like an informal focus group), we will use questions (e.g., What was your overall experience during the workshop?”) to guide the improvement of future digital storytelling workshops. We will audio record (MP4) this session.  **Incentives:** We will compensate each participant $300 ($150 per day) and provide childcare vouchers to participate in the two-day workshop.  **Deliverable for Aim 1:** The ten produced digital stories reflecting participants’ experiences of their children’s COVID-19 vaccination will serve as Aim 2 intervention materials.  **These videos are not finalized. We will add links to these videos to our IRB as a modification when they are finalized.  **We will also pilot reviewing the videos and Narrative Quality Assessments to present these at the CAB meeting (to select the videos we will use, described below).  **Data collected for Aim 1**: We will collect participants’ demographic information, the video recordings from the digital storytelling workshop, the final digital stories, and an evaluation after the workshop. We will keep this data for up to five years.  **Preparation for Aim 2:** Our CAB will review all digital stories and select the **four** most persuasive and culturally-relevant stories to use in our intervention. CAB members will complete a Narrative Quality Assessment tool (identification and transportation, α = 0.94, survey developed in prior storytelling studies with Hispanic participants by Co-I Larkey and validated by MPI Kim and to identify elements of stories that influence attitude and behavior change) immediately after reviewing each story. See our MOU for CAB members. If needed, the research team will edit the digital stories before using them in the intervention.  **AIM 2**:  **Intervention Delivery Procedures:** Community health workers will help us identify Hispanic parents whose child(ren) is/are not up-to-date with COVID-19 vaccine doses. They will provide the study team with contact information for these parents.  Participants will receive an email with a link that directs them to the T1 assessment, administered online using the REDCap data collection and management platform. If needed, we will ask CHWs to help schedule a time to help participants complete the surveys and intervention. Our CHWs have noted that sometimes they need to help individuals with limited literacy skills complete assessments. After participants complete the T1 assessment (baseline COVID-19 vaccine perspectives survey), we will randomize them to the intervention or control group. We will show the intervention group participants the four digital stories delivered in randomly assigned, counterbalanced orders. Immediately after viewing each story, intervention participants will be asked to rate the story using the Narrative Quality Assessment tool. This tool asks participants to rate the degree to which they identify with the story narrator and the story. After participants have watched all digital narratives and completed their corresponding Narrative Quality Assessment, we will ask them to complete the T2 assessment. This survey asks participants about their COVID-19 vaccine perspectives. Control group participants will receive a CDC COVID-19 Vaccine Information Sheet for their child’s age before completing the T2 assessment. All participants (intervention and control) will receive a resource kit containing information about where to obtain vaccines (e.g., walk-in clinics) so they do not need to wait for the child’s annual check-up to receive the vaccine from their regular provider.  Two months later, we will contact all participants and ask them to complete another follow-up (T3) assessment to assess participants’ vaccine hesitancy, intentions to vaccinate, and behaviors of vaccinating their children against COVID-19 have changed since T1 and T2.  **Incentives in Specific Aim 2:** We will ask parents who report vaccinating their child against COVID-19 at T3 to upload a photo of the child’s COVID-19 vaccine card. This is being collected as a more objective (than self-reported data) measure to assess behavior change (vaccine uptake).  We will compensate all participants $20 for each survey they complete for the study.  Additionally, we will conduct four focus groups (of 6-8 Hispanic parents each) with a sample of **intervention group participants**. We will recruit a stratified sample of intervention participants, including children who received COVID-19 vaccines since T2 and those who remained unvaccinated by T3, to participate in post-intervention focus groups. In these focus groups, we will explore their perceptions of the DST intervention and its impact on their decisions to vaccinate their children. We will compensate all focus group participants $25. Focus groups can last up to one hour.  Data Collection for Aim 2 Summary:  1) Intervention and Control Groups, T1 survey : Demographic survey, COVID-19 vaccine perceptions survey, vaccine hesitancy surveys . (Estimated time: up to 15 minutes)  2)Intervention group participants only: Narrative Quality Assessment per digital story viewed (Estimated time: up to 20 minutes)  3) Intervention and Control Groups, T2 survey: COVID-19 vaccine perceptions survey + vaccine hesitancy survey  4) Intervention and Control Groups, T3: Assess whether children received any COVID-19 vaccine doses two months post-intervention  5)*Select group of intervention participants - one-time online (recorded) focus group about the study | |
| **Withdrawal of Participants** | |
| There are no anticipated circumstances under which participants will be withdrawn from the research study without their consent.  If participants choose to no longer take part in this study, they are asked to contact one of the study PIs (Alexis Koskan, [alexis.koskan@asu.edu](mailto:alexis.koskan@asu.edu); 480-884-2533, Extension 42533) to notify her that they will no longer be involved in the study. She will remove and destroy all participant data from the study. | |
| **Risks to Participants** | |
| We do not anticipate any other psychological, legal, or social risks resulting from this study.  **Physical risk:** No physical risks are associated with participation in this study.  **Psychological risk**: Given that the topic of COVID-19 vaccines has been a polarizing health issue, telling personal stories or viewing videos that present stories about COVID-19 vaccines may make participants feel anxious. Some participants may experience `. Our informed consent document will tell participants that their participation is voluntary, and they can skip any questions they prefer not to answer. In addition, the informed consent document will state that the data collected for this study will be strictly confidential. We will not share participants’ responses with anyone else to the extent provided by law (e.g., child abuse, self-harming, or intent to hurt someone else).  In prior research, we tested the discussion guide and the measures used for the DST workshop and intervention. We are updating these materials to reflect COVID-19 vaccines. No participants reported adverse reactions to the discussion guide or study measures in our previous digital storytelling research. The research team will monitor participants carefully for any adverse effects of participating in the study during the study period.  **Breach of participant confidentiality** is the most significant risk in the proposed study. We describe our risk protection plan in Section 16, Data Management and Confidentiality. | |
| **Potential Benefits to Participants** | |
| Participants recruited for Aim 2 activities may benefit from this study by learning more about COVID-19 vaccines and COVID-19 vaccination as a prevention strategy. Participants for Aim 2 (and the exploratory aim) may also benefit from the study by increasing their intention to vaccinate their children against COVID-19 and consequently promote their children’s health.  Additionally, all study participants will receive incentives to compensate them for their time and efforts. These benefits outweigh any potential risks of participation. | |
| **Setting** | |
| **Aim 1 Setting:** We will execute planned activities through an in-person workshop to guide our participants to develop digital stories (each 2-3 minutes long) through a process of ideation, development, production, post-production, and digital distribution. A certified DST workshop facilitator, MPI Kim, will conduct the online DST workshops during two consecutive 8-hour days.  The CAB meeting that will follow the creation of these videos (when CAB members select the four most persuasive and relevant digital stories) will take place online unless the CAB prefers meeting in person. We will leave this decision as optional based on the CAB members’ preferences. We will update our protocol/IRB submission accordingly. This is not a formal focus group.  **Aim 2 Setting**: The intervention will fully be delivered online. In this study, participants will receive an email with a link that directs them to the T1 assessment, administered online using REDCap. If participants need assistance to complete the study, we will help them schedule a time to complete the study with one of our three CHWs. After participants complete the T1 assessment, we will randomize them to the intervention or control group. We will show the intervention group participants the four selected digital stories. We will deliver the stories in randomly assigned, counterbalanced orders. Immediately after viewing each story, intervention participants will be asked to rate the story using the Narrative Quality Assessment Tool. This tool, which has six items, asks participants to rate how they identify with the story narrator and the storyline. After participants have watched the three digital narratives and completed their corresponding Narrative Quality Assessment, they will complete the T2 assessment. For T3, we will text participants and ask them whether their child received any additional COVID-19 vaccine doses two months after the completed the intervention.  Later, we will **conduct four online focus groups**, each with 6-8 intervention group participants. | |
| **Multi-Site Research** | |
| Not Applicable | |
| **Resources Available** | |
| This study uses standard educational resources (internet, Zoom, online survey software) to complete the research. | |
| **Prior Approvals** | |
| This funding for this study (NIH R21) has been awarded.  We have an MOU (uploaded) for our community health worker organization (help with study recruitment and dissemination of findings see Other Attachments). | |
| **Data Management and Confidentiality** | |
| All information and documents completed by research participants will be used only for research purposes and will be strictly confidential. Where possible, we will transfer all electronic data to REDCap software and store all data on a secure server. As data entry concludes for the pre-and post-intervention data collection waves, we will copy the completed files to removable media (CD); one copy will be kept secure in a locked drawer of the PI’s locked office. This information is meaningless to anyone not associated with the project. We will remove all identifying information and any data shared with other institutions and researchers under NIH data sharing guidelines (aside from general demographic information—ethnicity, age, gender).  How we will reduce study risks:  We will take additional steps to protect the confidentiality of participants’ data:  All study personnel will be CITI certified.   - We will generate a list of authorized personnel (who can handle data). We will only allow those study team members access to the data. - All study personnel will sign an Assurance of Confidentiality form. Breach of confidentiality will be grounds for immediate termination. - Study personnel will not be allowed to abstract data or process data from any participants they know personally. - Informed consent documents will be stored online on password-protected, locked computer databases (REDCap). - To keep information confidential, we will assign a study ID number when participants are enrolled. We will keep a master list of their ID numbers and identifying information (name, contact information, study ID number) separately in a secured, locked database on a password-protected computer kept in a locked office. We will keep participants identifying information in this master list until after the T3 data collection point. At that time, we will destroy all identifying information. Participants will be able to withdraw from the study at any time, and their data will not be used for the study – until the master list has been destroyed. - All survey data will be stored in our University’s secure server where it can only be assessed by our research staff. Study data will not be shared with other researchers or industry partners for future research or other uses. - This clinical trial will be described on http://www.ClinicalTrials.gov, as U.S. Law requires. This website will not include any identifying information. - We will keep all study data (other than identifying information) for up to five years before we destroy it. - We will utilize screensaver passwords on all team members’ computers to prevent access to data while individuals are out of the office. - We will enter children’s COVID-19 vaccine uptake data as soon as we receive the photos of their COVID-19 immunization records. We will delete the photos immediately after entering vaccine uptake data into our secure database. | |
| **Safety Monitoring** | |
| The study Data Safety and Monitoring Board and, separately, the research team will review all study data for completeness, abidance of the study protocol; potential complaints by participants; recruitment, enrollment, and retention of participants; participant confidentiality and privacy; safety from any risks (foreseen and unforeseen). | |
| **Consent Process** | |
| All participants interested in enrolling in the study must complete an informed consent document. Our informed consent document will include the following key elements:  (1) the purpose of the research;  (2) the risks and benefits associated with participation in the research;  (3) the confidentiality of responses to the research instruments and in data collected from medical records;  (4) the stipulation that participation in the study is voluntary; and  (5) the stipulation that withdrawal from the study and refusing to answer specific questions are options during study participation and that no negative consequences will result from these actions.  Participants in Specific Aim 1 will be asked to complete a Study Release Form, allowing the research team to use the digital story they create for Specific Aim 2 of the study.  All participants in Aim 1 will be asked to sign a copy of the informed consent form. We will give each participant a copy of the informed consent should they have any questions or concerns about the study. We will store all study informed consent documents (with their signatures) on an encrypted database (REDCap) on a password-protected computer.  In Aim 2, participants will signify consent by completing study surveys. The consent form in this aim provides information about the potential for participants to be re-contacted to participate in post-intervention focus groups. | |
| **Investigational New Drug or Devices** | |
| NA | |
| **CITI**  Provide the date that the members of the research team have taken the CITI training for human participants. This training must be taken within the last 4 years. Additional information can be found at: <http://researchintegrity.asu.edu/training/humans> | |
| CITI certification for Koskan, Kim, Larkey, and Todd can be found at ASU’s IRB. | |
|  | |
